# Agreement between Capillary Refill Time measured at Finger and Earlobe sites in different positions: a pilot prospective study on healthy volunteers

**DOI:** 10.1186/s12871-022-01920-1

**Published:** 2023-01-18

**Authors:** Luigi La Via, Filippo Sanfilippo, Carlotta Continella, Tania Triolo, Antonio Messina, Chiara Robba, Marinella Astuto, Glenn Hernandez, Alberto Noto

**Affiliations:** 1 Department of Anesthesia and Intensive Care Medicine, Azienda Ospedaliero Universitaria “Policlinico – San Marco”, 95123 Catania, Italy; 2 School of Specialization in Anesthesia and Intensive Care, University of Catania, 95123 Catania, Italy; 3 School of Specialization in Anesthesia and Intensive Care, University Magna Graecia, 88100 Catanzaro, Italy; 4 Department of Anesthesia and Intensive Care Medicine, Humanitas Clinical and Research Center-IRCCS, 20089 Rozzano, Milan, Italy; 5 Anesthesia and Intensive Care, IRCCS for Oncology and Neurosciences, San Martino Policlinico Hospital, 16100 Genoa, Italy; 6 Departamento de Medicina Intensiva, Facultad de Medicina, Pontificia Universidad Católica de Chile, Santiago, Chile; 7 Division of Anesthesia and Intensive Care, University of Messina, Policlinico’’G. Martino’’, 98121 Messina, Italy

**Keywords:** Perfusion, Hemodynamics, Accuracy, Precision, Intensive care, Critical illness

## Abstract

**Background:**

Capillary Refill Time (CRT) is a marker of peripheral perfusion usually performed at fingertip; however, its evaluation at other sites/position may be advantageous. Moreover, arm position during CRT assessment has not been fully standardized.

**Methods:**

We performed a pilot prospective observational study in 82 healthy volunteers. CRT was assessed: a) in standard position with participants in semi-recumbent position; b) at 30° forearm elevation, c and d) at earlobe site in semi-recumbent and supine position. Bland–Altman analysis was performed to calculate bias and limits of agreement (LoA). Correlation was investigated with Pearson test.

**Results:**

Standard finger CRT values (1.04 s [0.80;1.39]) were similar to the earlobe semi-recumbent ones (1.10 s [0.90;1.26]; *p* = 0.52), with Bias 0.02 ± 0.18 s (LoA -0.33;0.37); correlation was weak but significant (*r* = 0.28 [0.7;0.47]; *p* = 0.01). Conversely, standard finger CRT was significantly longer than earlobe supine CRT (0.88 s [0.75;1.06]; *p* < 0.001) with Bias 0.22 ± 0.4 s (LoA -0.56;1.0), and no correlation (*r* = 0,12 [-0,09;0,33]; *p* = 0.27]. As compared with standard finger CRT, measurement with 30° forearm elevation was significantly longer (1.17 s [0.93;1.41] *p* = 0.03), with Bias -0.07 ± 0.3 s (LoA -0.61;0.47) and with a significant correlation of moderate degree (*r* = 0.67 [0.53;0.77]; *p* < 0.001).

**Conclusions:**

In healthy volunteers, the elevation of the forearm significantly prolongs CRT values. CRT measured at the earlobe in semi-recumbent position may represent a valid surrogate when access to the finger is not feasible, whilst earlobe CRT measured in supine position yields different results. Research is needed in critically ill patients to evaluate accuracy and precision at different sites/positions.

## Introduction

Capillary Refill Time (CRT) is a marker of peripheral perfusion and an indicator of sympathetic activation. Its use has been suggested in critical scenarios and in the case of impaired tissue perfusion [1–4]. Of note, the use of CRT is characterized by important advantages. Indeed, it is easy to perform at the patient's bedside, and its assessment is not time-consuming (lasting less than one minute); although several instruments have been tested for automated measurement [5–9], CRT does not necessarily require expensive devices or equipment, nor sampling of patient’s blood. Moreover, CRT seems rapidly responsive to resuscitation therapy with fluids or vasopressors and can be repeated to monitor effects of therapies. For all these reasons, CRT could be considered an attractive method to guide management and therapy in patients admitted to intensive care unit (ICU), in particular during the initial phase of critical illness, in combination or as an alternative to other methods (i.e. lactate measure). Not surprisingly, prolonged CRT has been correlated with signs of organ failure and mortality in ICU patients. Of note, CRT was recently included in the international guidelines for the management of septic shock [10].

Recently, the ANDROMEDA-SHOCK trial found that patients in the CRT group had an almost significant reduction in 28-day mortality and lower SOFA scores at 72 h [11], thus the doors to a novel approach to sepsis tailored on the use of CRT, which is currently under investigation with the multicenter randomized ANDROMEDA-SHOCK 2 trial (registered on www.clinicaltrial.gov NCT05057611) [12].

A couple of methodological criticalities should be taken into account when adopting CRT measurements. First, standard CRT measurement at the finger level cannot be easily used in the operating room, because of the difficulty to access the patient’s hands. Evaluation of CRT at a different and more accessible (and compressible) anatomic site may be advantageous in such cases, since intraoperative hypotension, which can lead to hypoperfusion, has been associated to increased risk of postoperative organ injury [13–15]. Considering that most surgical interventions are conducted with unrestricted access to the patient’s head, the earlobe could be a valid option, but this option has not been yet explored. A second methodological issue is represented by the position of patient’s arm when performing CRT measurement. In fact, CRT depends on blood flow which should be described as (arterial pressure – critical closing pressure)/resistance [16]. In this context, it has been suggested that the position and height of the patient’s hand may alter the critical closing pressure and/or the resistances, in turn affecting CRT values [17]. Moreover, it is possible that the elevation of the hand position may favor venous return and reduce CRT measurements. However, whether this change in hand position truly affects the CRT results has not been properly addressed.

Therefore, in this pilot study we aimed at addressing the above-described methodological criticalities. In particular, we hypothesized that CRT measurements taken at finger in standard approach could be interchangeable to those detected at the earlobe site, whilst CRT values measured at the finger level in different anatomical positions would be different.

## Materials and methods

We performed a pilot prospective observational study in healthy volunteers, involving personnel of the School of Anaesthesia and Intensive Care of the University of Catania and staff of the General ICU of the Azienda Ospedaliera Universitaria “Policlinico-San Marco”, Catania. The study was approved on the 21/03/2022 by our local Ethical Committee (“*Comitato Etico Catania 1*”—Reference protocol: 52/2022/PO).

### Participants

We included healthy adult volunteers regardless of age and gender. Participants were instructed to breath normally during the examination. Rings and earrings were removed before the procedure. Exclusion criteria were missing informed consent, hands and/or all fingers amputation, diagnosis of peripheral vascular disease, burn injuries to hands and fingers and face.

### Study interventions

The CRT measurement is a routine practice in our ICU and its normal value is reported to be ≤ 2 s [1]. CRT evaluation was standardized as follows [11, 18]:

A) Pressure application by the operator on the distal phalanx of the patient index, on the non-nailed side, with the use of a small microscope transparent slide, which allows adequate skin color visualization. Such pressure is applied continuously for 15 s, observing the skin blanching..
B) After 15 s, pressure is released and the operator measures the seconds needed for the return of basal skin color, which is the time taken to refill again the peripheral capillaries. Such time is defined as CRT.

Each procedure was performed by the same assessor (LLV) who was in charge of pressure application and release, and of CRT evaluation. A second operator was responsible of recording all the measurements with a camera and using a stopwatch as follows: the second operator started and stopped the stopwatch at the first operator’s commands of “start” (pression released) and “stop” (skin color normalization). The CRT value was defined as the time between the command “start” and the command “stop”. This value was collected by the second operator in real time and it was not communicated to the assessor (LLV) until all the measurements for each volunteer were taken. This procedure was adopted to reduce the risk of measurement error, ensuring the assessor was free to focus only on the evaluation of skin color.

We performed four type of CRT measurement in a randomized order, with a casual simple sampling, through the identification of the order according to the extraction from sealed bags. There were 4 sealed bags for each participant, each containing a number that identifies the measurement to do. Between measurements, a 30-s period of resting was applied.

**Fig. 1:**
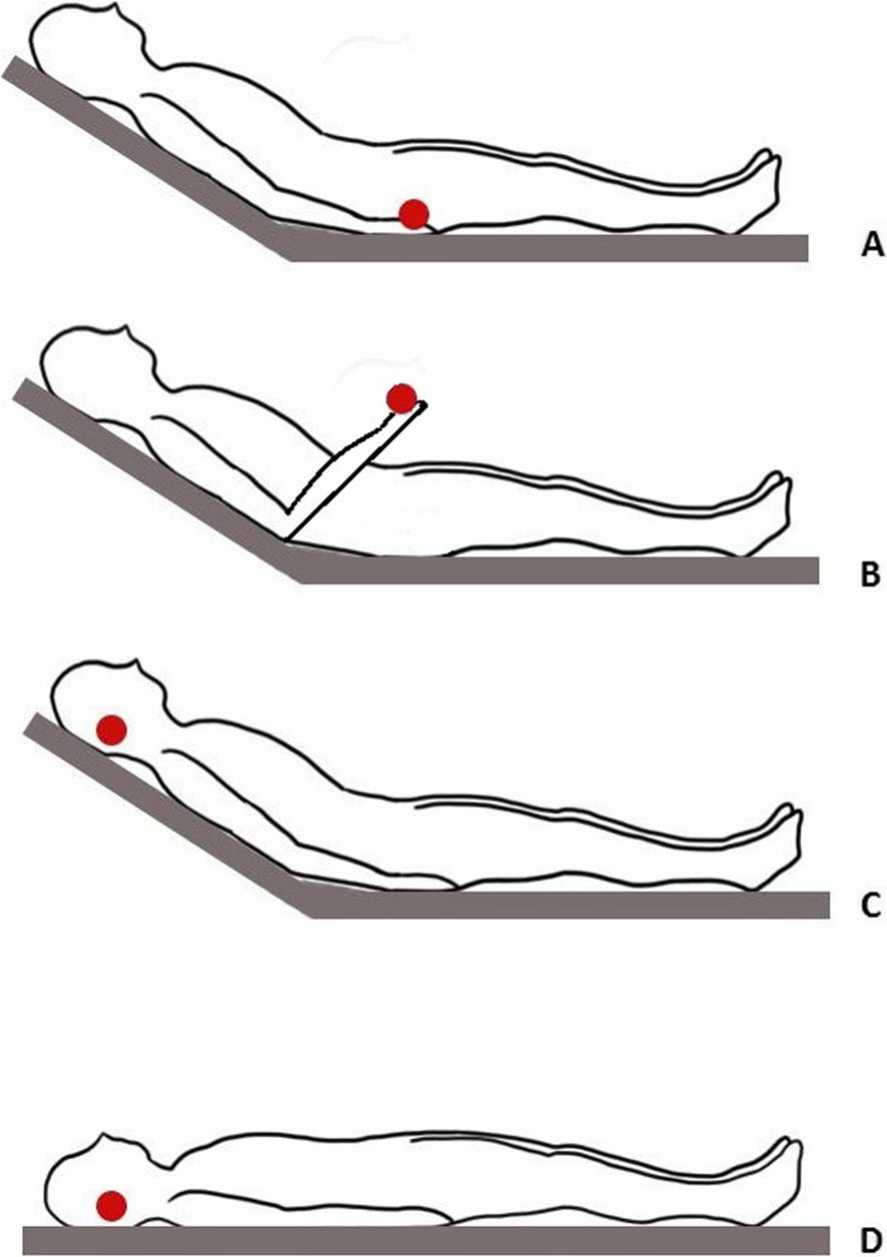
CRT measurement positions. **A**: standard finger measurement; **B**: elevated finger measurement; **C**: earlobe semi-recumbent measurement; **D**: earlobe supine measurement

As shown in Fig. 1, we undertook four CRT measurements. The first three were performed with the volunteer in semi-recumbent (30°-35°) position: a) *standard finger measurement* performed at the index with the arm lying flat on the bed; b) *elevated finger measurement* performed after forearm elevation of 30° (hand positioned roughly at the right atrium level); c) *earlobe semi-recumbent measurement* with CRT measured at earlobe. The fourth CRT measurement was performed at the earlobe with the participant in supine position (*earlobe supine measurement)*. Thus, each participant received a single measurement for each of the four area/position. The volunteers maintained their position for one minute before each measurement.

From each participant we recorded baseline anthropometric data, body temperature and SpO2, as well as hemodynamic variables using Clearsight® noninvasive monitoring; in particular we registered baseline cardiac output, stroke volume, heart rate and arterial pressure (systolic, diastolic, mean).

### Outcomes of the study

We primarily compared the CRT measurement performed at the standard finger position (arm lying in bed with the volunteer in semi-recumbent position) with:

1. those registered at the earlobe site in the two bed positions (semi-recumbent and supine).
2. CRT measurements performed at patient’s finger in elevated position with raised hand and forearm.

### Statistical analysis

Two recent studies showed values of CRT in healthy volunteers ranging between 1.12 and 1.37 s [5, 8], Therefore, the sample size was calculated considering a mean CRT value for standard finger measurement of 1.25 s, with a standard deviation of 0.4 s. We hypothesized a CRT reduction of 0.25 s with measurements performed in *elevated finger* and *earlobe semi-recumbent* due to facilitation in venous return. Thus, assuming a statistical power of 80% and an α level of significance at 0.05, the sample size calculation suggested enrolling 80 healthy volunteers. Considering up to a maximum of 10% for missing data *(n* = *8)*, we planned to enroll 89 adult healthy volunteers.

Data were reported as numbers (percentages) for the categorical variables, and as mean (and standard deviation—SD) or median (and interquartile range – IQR) for the continuous variables according to the distribution that was verified with Kolgomorov-Smirnoff test. The univariate analysis for continuous variables were conducted using the Student t-test for paired samples if data were normally distributed, or with the corresponding non-parametric test in case of non-normally distributed data. The univariate analysis for categorical variables were conducted using the Fisher’s exact test. The relation among 2 variables will be evaluated by calculating the correlation coefficient of Pearson. We calculated the agreements (bias, SD and limits of agreement [LoA]) between CRT measurement in different areas/modalities with the Bland and Altman test for repeated measures. The bias indicates the accuracy of measurements methods, while the LOA specifies the precision.

## Results

Data were collected from 88 young volunteers, but we excluded two volunteers as they had a diagnosis of Raynaud’s syndrome and four volunteers as they had cold hands even after attempts to warm up with hot water. All anthropometric and hemodynamic variables are reported in Table1, together with the results ofCRT measured in the 4 conditions.

**Table 1:** Baseline characteristics of the healthy volunteers participating in the study and values of capillary refill time (CRT) in different sites and positions

VARIABLE	Value
Age, median [IQR], years	30 [27–35]
Weight, median [IQR], kg	65 [57–79]
Height, mean (SD), cm	169 (8.3)
Heart Rate, mean (SD), bpm	77 (10)
Systolic Arterial Pressure, mean (SD), mmHg	108 (13)
Mean Arterial Pressure, mean (SD), mmHg	83 (10)
Diastolic Arterial Pressure, mean (SD), mmHg	68 (8)
Cardiac Index, mean (SD), L/min	4.36 (1.19)
Temperature, mean (SD), °C	36.5 (0.2)
Oxygen saturation with pulse-oximetry, median [IQR], %	98 [97–99]
Respiratory Rate, median [IQR] bpm	13 [12, 13]
**CRT MEASUREMENTS**	**Value**
*Standard Finger* CRT, median [IQR], sec	1.04 [0.8–1.39]
*Elevated Finger* CRT, median [IQR], sec	1.17 [0.93–1.41]
*Earlobe Semi-recumbent* CRT, median [IQR], sec	1.10 [0.90–1.26]
*Earlobe Supine* CRT, median [IQR], sec	0.88 [0.75–1.06]

*IQR* Interquartile range, *SD* Standard deviation

**Fig. 2:**
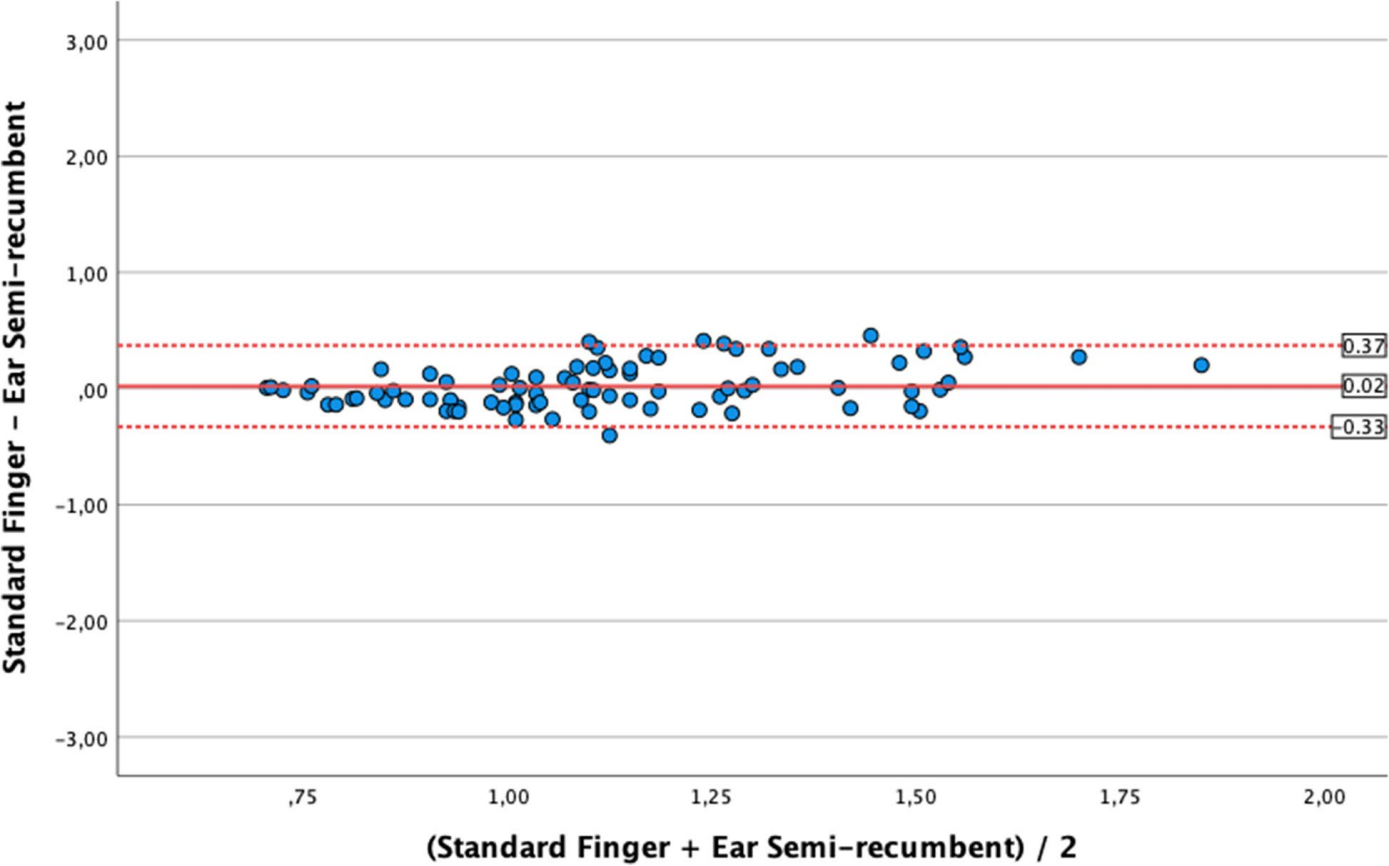
Bland Altman analysis for the comparison of values of capillary refill time (CRT) obtained at standard finger position as compared to earlobe semi-recumbent measurements

As shown in Fig. 2 (Bland–Altman diagram), median values of *standard finger CRT* (1.04 s [IQR 0.8–1.39]) were similar to the median *earlobe semi-recumbent CRT* (1.10 s [IQR 0.90–1.26], *p* = 0.52). The agreement analysis showed a mean Bias of 0.02 ± 0.18 s with LoA between -0.33 s and 0.37 s. Pearson analysis showed a significant but weak correlation (*r* = 0.28 [0.07;0.47]; *p* = 0.01).

**Fig. 3:**
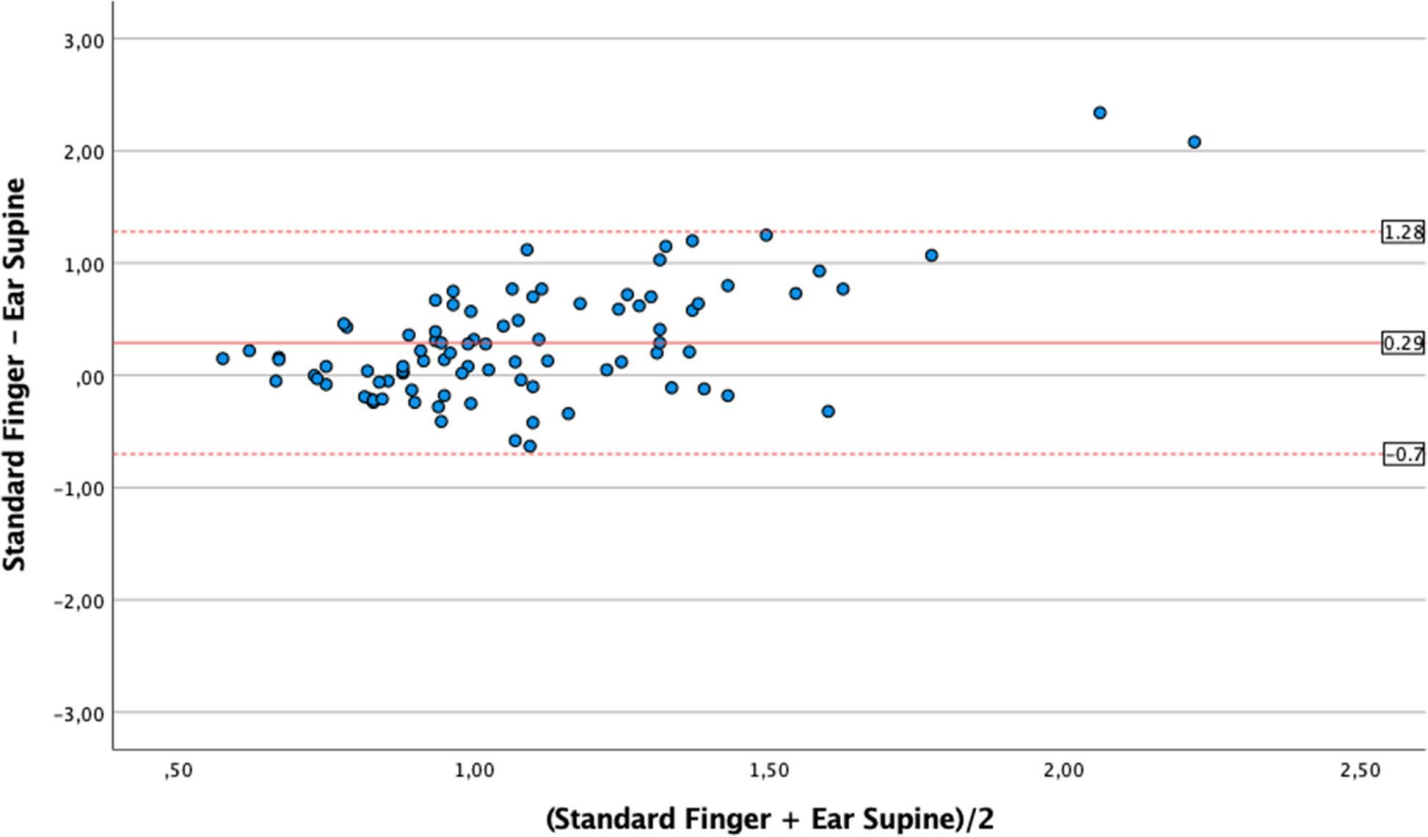
Bland Altman analysis for the comparison of values of capillary refill time (CRT) obtained at standard finger position as compared to earlobe supine measurements

Conversely, median *standard finger CRT* was significantly longer than median *earlobe supine CRT* (0.88 s [IQR 0.75–1.06], *p* < 0.001). The agreement analysis showed a mean Bias was 0.22 ± 0.4 s and LoA were -0.56 to 1.0 s (Fig. 3). The correlation was not significant (*r* = 0.12 [-0.09; 0.33];*p* = 0.27).

**Fig. 4:**
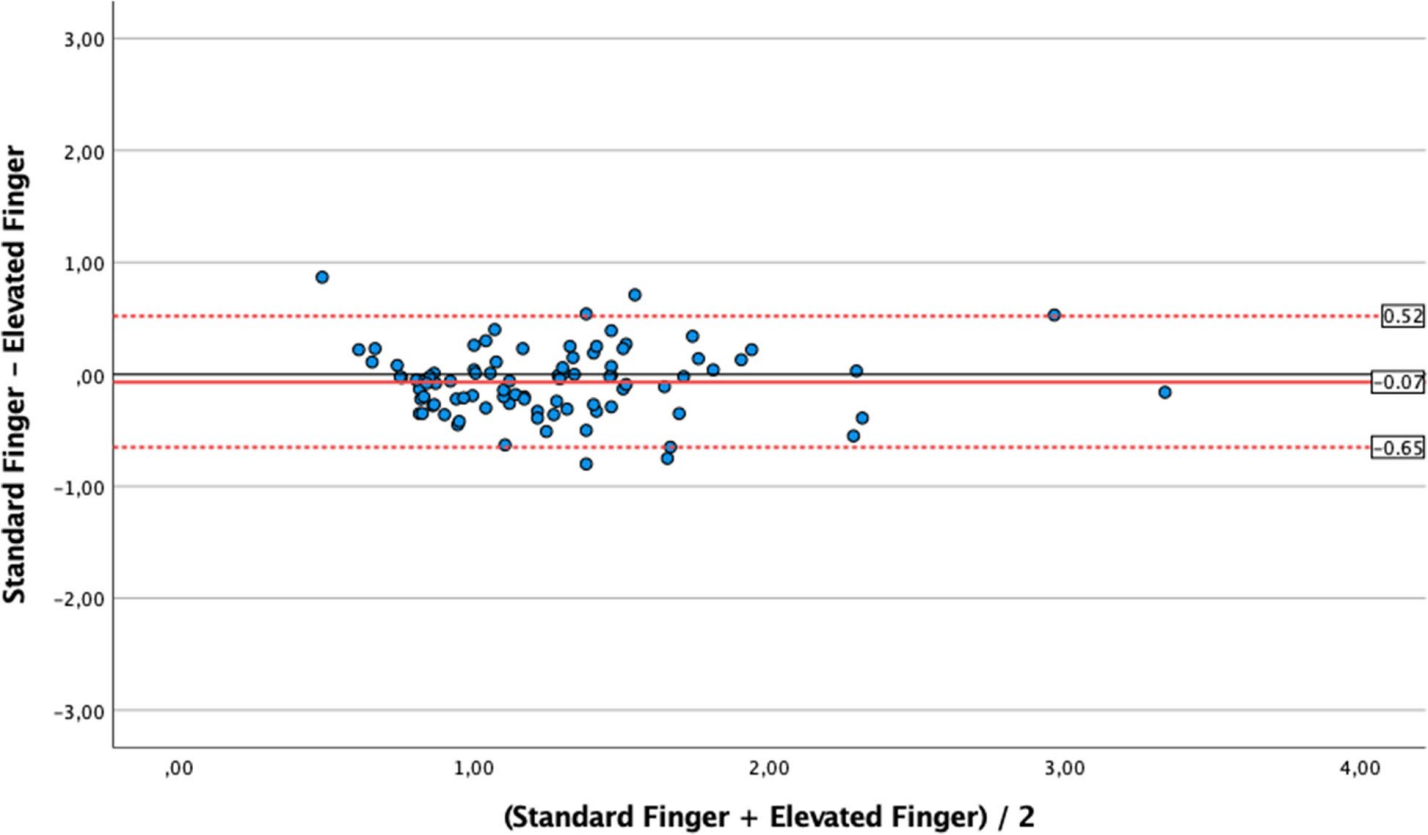
Bland Altman analysis for the comparison of values of capillary refill time (CRT) obtained at standard finger position as compared to elevated finger measurements

The median *elevated finger CRT* (1.17 s [IQR 0.93–1.41] was significantly longer than median *standard finger CRT* (*p* = 0.03); as shown in Fig. 4, there was a mean Bias of -0.07 ± 0.3 s between these measurements with LoA of -0.61 (lower) and 0.47 (upper). Pearson analysis showed a significant correlation of moderate degree (*r* = 0.67 [0.53–0.78], *p* < 0.001).

All the results of CRT measurements and their comparison between median, mean Bias and LoA, and correlation are reported in Table 2. For completeness all the possible comparisons between CRT measurements are shown.

**Table 2:** Analyses of Capillary Refill Time measurements at different sites and position. For each comparison we report the median difference (non-parametric test for paired samples), the agreements (bias and standard deviation with limits of agreement [LoA]), and the Pearson correlation coefficient (in italic)

	Standard Finger	Elevated Finger	Ear Semi-recumbent	Ear Supine
Standard Finger		*p* = 0.03	*p* = 0.52	*p* < 0.001
		Bias -0.07 ± 0.30 LoA -0.61; 0.47	Bias 0.02 ± 0.18 LoA -0.33; 0.37	Bias 0.22 ± 0.40 LoA -0.56; 1.0
		*r* = *0.67 [0.53;0.78]; p* < *0.001*	*r* = *0.28 [0.07;0.47]; p* = *0.01*	*r* = *0.12 [-0.09;0.33]; p* = *0.27*
Elevated Finger	*p* = 0.03		*p* = 0.12	p < 0.001
	Bias -0.07 ± 0.30 LoA -0.61; 0.47		Bias 0.12 (SD 0.33) LoA -0.52; 0.76	Bias 0.29 ± 0.39 LoA -0.47; 1.05
	*r* = *0.67 [0.53;0.78]; p* < *0.001*		*r* = *0.37 [0.17;0.54]; p* < *0.001*	*r* = *0.08 [-0.13;0.29]; p* = *0.46*
Ear Semi-recumbent	*p* = 0.52	*p* = 0.12		*p* < 0.01
	Bias 0.02 ± 0.18 LoA -0.33; 0.37	Bias 0.12 ± 0.33 LoA -0.52; 0.76		Bias -0.17 ± 0.26 LoA -0.67; 0.33
	*r* = *0.28 [0.07;0.47]; p* = *0.01*	*r* = *0.37 [0.17;0.54]; p* < *0.001*		*r* = *0.40 [0.20;0.57]; p* < *0.001*
Ear Supine	*p* < 0.001	*p* < 0.001	*p* < 0.01	
	Bias 0.22 ± 0.40 LoA -0.56; 1.0	Bias 0.29 ± 0.39 LoA -0.47; 1.05	Bias -0.17 ± 0.26 LoA -0.67; 0.33	
	*r* = *0.12 [-0.09;0.33]; p* = *0.27*	*r* = *0.08 [-0.13;0.29]; p* = *0.46*	*r* = *0.40 [0.20;0.57]; p* < *0.001*	

## Discussion

In this pilot study on healthy volunteers, we primarily compared CRT at standard hand level in semi-recumbent position with CRT measurements registered at the earlobe site in two bed positions (semi-recumbent and supine), or with the measurement of CRT in a different forearm/hand position. The main findings of our study were: 1) as compared to the standard finger position, CRT measured at the earlobe site produced similar results with participants lying in semi-recumbent (*p* = 0.52, bias 0.02 ± 0.18 s; LoA -0.33;0.37) but not in the supine position (*p* < 0.001, bias 0.22 ± 0.4 s; LoA -0.56;1.00); 2) significant differences in the CRT measured in the two different forearm/hand positions (standard or elevated finger, *p* = 0.03), with small bias (-0.07 ± 0.3 s) but large LoA (-0.61;0.47). Most of the correlations between measurements were weak as they had a non-linear trend. Overall, our results suggest that earlobe in supine position produces different results as compared to the standard finger evaluation, whilst more reproducible findings are obtained with the earlobe semi-recumbent position. Therefore, our hypothesis of using the earlobe site in supine position as possible surrogate of the standard finger CRT measurement does not seem supported. Nonetheless, when performing surgery with the patient in semi-recumbent position, the earlobe site may become a more attractive surrogate site for performing CRT. The second result suggests that the position of the finger when performing CRT calculation is an important issue. Whether the differences we showed are greater in an elderly people and/or in critically ill patients remains to be established, and our group is currently investigating this issue. Nonetheless, it is likely that standardization of the measurement of CRT at finger level becomes crucial in clinical studies, in consideration of the likelihood of differences whilst changing the hand position.

Saito et coworkers [17] recently published interesting results, demonstrating a CRT variation between two positions (recumbent vs sitting) and between different hand elevations. Their analysis showed that the elevation of the hand to the heart level prolongs the CRT value. Similarly, elevation of the forearm/hand in our study produced an increase in the CRT measurements that seems more related to a reduction in arterial pressure next to the site of measurement due to hydrostatic arterial pressure drop than to venous return (stasis) phenomena. In this regard, it is also worth noting that the change of bed position produced significant variations also at the other anatomical site with a bias -0.17 s and wide LoA (-0.67; 0.33) measured at earlobe level in supine or semi-recumbent position. Therefore, our findings contradict the initial hypothesis where we expected that the elevation of the hand would have facilitated venous return and decreased congestion, in turn fastening the capillary refill. It is possible that in healthy volunteers, where congestion is not an issue and the cardiac function is normal, the position of the forearm/hand has a greater impact on the perfusion pressure (afterload) rather than on the venous return. The differences in CRT measurements with different forearm/hand position seems small, but whether these variations may become larger and clinically meaningful under condition of shock with hypotension and hypo-perfusion remain an open research question. Moreover, it is also possible that cardiac dysfunction may influence the results at different positions.

CRT remains a useful physical examination to explore adequacy of peripheral perfusion during a state of any type of shock. Although this technique was proposed by Beecher et al. in 1947 [19], to date, no specific guidelines exist to standardize the execution of CRT measurement. The CRT assessment could be affected by many factors: skin color, ambient temperature, age, ambient light, duration and strength of pressure [20–24]. In this regard, our results apply to a Caucasian healthy young population and may not be applicable to the elderly, to other races or in case of impaired perfusion [25–27]. Although clinicians should always be aware of these confounding factors, the ANDROMEDA-SHOCK trial [11] demonstrated that CRT could be as effective as conventional markers of peripheral perfusion in tailoring resuscitation therapies. Improvements in CRT technology with devices applying standardized pressure and performing objective measurements could be the optimal solution [28], but this would generate healthcare costs related to the device acquisition. Also, a recent cross-sectional survey performed on French intensivist showed that only 3% of the responders used a chronometer to assess CRT [29]. In this regards, specific training on CRT use could be a possible way to reduce the great variability of CRT assessment [30]. In our opinion, it is mandatory to collect more data and knowledge on the CRT at different sites and producing a standardization of the technique that reduce inter-observer variability.

### Limitations

This study has several limitations that should be considered when interpreting the results. The main limitation is that we only studied a sample of healthy young subjects without extreme values, which may be required to evaluate the agreement between the two methods. It is likely that differences in patients with shock could be much larger. However, it should be also noted that each measure was repeated in all the healthy volunteers and this reduces the intrinsic variability of CRT (age, skin color, ambient temperature, etc.). Moreover, measurements were performed by the same trained research operator with the aid of a second operator dealing with camera and stopwatch, so that the assessor was focused on CRT evaluation only. However, we did not perform the external validation of the measurements with the analysis of the videos by a blinded operator, thus leading to a possible source of measurement errors. Second, we acknowledge that this approach could have been improved by an off-line random evaluation of the videos recorded, as previously described [31, 32], and eventually with the reproduction mode in slow motion. Off-line evaluation would have avoided also any influence on the assessor by previous CRT values. However, we decided for a more practical allowing real-time data collection for some reasons: 1) we expected very short CRT values (in our study averages ranged between 0.9 and 1.2 s) and such small times are unlikely to bias subsequent measurements; 2) the assessor did not know the CRT values until all the measurements were taken from each volunteer; 3) we intended to maintain a pragmatic approach reproducible from clinical perspectives. Nonetheless, off-line review would be certainly a valuable option when dealing with critically ill patients with prolonged CRT in future studies. Third, although our hypotheses were partly rejected, the difference is relatively small. The presence of differences in the measurement of CRT (i.e. accuracy at different sites/positions) does not rule out its possible usefulness. Indeed, precision and trending ability are probably the most important characteristics of markers of perfusion, where trends are relevant if they move in the same direction in response to treatments and variation of the clinical conditions. Our data do not support equivalence of measurements but they do not rule out the clinical implementation with different cut-offs. Fourth, although we did not use a device to standardize the pressure applied and the skin color evaluation, we performed this study under the same light conditions. Fifth, we studied healthy volunteers with a narrow range of CRT values and this may have decreased the probability to find correlations between different sites. Sixth, we did not evaluate the CRT at forehead, which is easy to perform and it seems to be less influenced by the ambient temperature compared to the fingertip [33].

## Conclusions

In a population of healthy volunteers, the elevation of the hand and forearm increases the CRT values measured at finger level. CRT measured at the earlobe in semi-recumbent position may represent a valid surrogate when access to the finger is not possible (i.e. during surgery), whilst CRT measured at earlobe in supine position yields different results. Clinical studies are needed to investigate changes of CRT in different anatomical sites and positions.

## Data Availability

The datasets used and/or analyzed during the current study are available from the corresponding author on reasonable request.

## Abbreviations

CRT: Capillary Refill Time
ICU: Intensive Care Unit
SD: Standard deviation
IQR: Interquartile range; LoA: limits of agreement
